# Understanding healthcare provider absenteeism in Kenya: a qualitative analysis

**DOI:** 10.1186/s12913-019-4435-0

**Published:** 2019-09-11

**Authors:** Katherine Tumlinson, Margaret W. Gichane, Siân L. Curtis, Katherine LeMasters

**Affiliations:** 1 Department of Maternal and Child Health, Gillings School of Global Public Health, University of North Carolina at Chapel Hill, 135 Dauer Drive, Chapel Hill, NC 27599 USA; 2 Carolina Population Center, University of North Carolina at Chapel Hill, 123 West Franklin Street, University Square, Chapel Hill, NC 27516-3997 USA; 3 Department of Health Behavior, Gillings School of Global Public Health, University of North Carolina at Chapel Hill, 135 Dauer Drive, Chapel Hill, NC 27599 USA; 4 Department of Epidemiology, Gillings School of Global Public Health, University of North Carolina at Chapel Hill, 135 Dauer Drive, Chapel Hill, NC 27599 USA

**Keywords:** Absenteeism, Quality of care, Kenya, Maternal and child health, Healthcare providers, Qualitative

## Abstract

**Background:**

Healthcare worker absenteeism is common in resource limited settings and contributes to poor quality of care in maternal and child health service delivery. There is a dearth of qualitative information on the scope, contributing factors, and impact of absenteeism in Kenyan healthcare facilities.

**Methods:**

In-depth semi-structured interviews were conducted between July 2015 and June 2016 with 20 healthcare providers in public and private healthcare facilities in Central and Western Kenya. Interviews were audio-recorded, transcribed, coded, and analyzed using an iterative thematic approach.

**Results:**

Half of providers reported that absenteeism occurs in both private and public health facilities. Absenteeism was most commonly characterized by providers arriving late or leaving early during scheduled work hours. The practice was attributed to institutional issues including: infrequent supervision, lack of professional consequences, limited accountability, and low wages. In some cases, healthcare workers were frequently absent because they held multiple positions at different health facilities. Provider absences result in increased patient wait times and may deter patients from seeking healthcare in the future.

**Conclusion:**

There is a significant need for policies and programs to reduce provider absenteeism in Kenya. Intervention approaches must be cognizant of the contributors to absenteeism which occur at the institutional level.

**Electronic supplementary material:**

The online version of this article (10.1186/s12913-019-4435-0) contains supplementary material, which is available to authorized users.

## Background

The last two decades have seen substantial political and financial commitments to improving the health of women and children living in low-income countries. Maternal and child health (MCH) is embedded in multiple Millennium and Sustainable Development Goals, is the focus of the United Nations Secretary-General’s Global Strategy for Women’s Health, and is on the national political agendas of many developing countries [1–3]. Globally, maternal mortality and under-five mortality have decreased 44 and 56% since 1990, respectively [3, 4]. Yet, maternal and child mortality remain high in many low-income settings within sub-Sarahan Africa. Sub-Saharan Africa accounts for less than 20% of the world’s population but 66 pecent of all maternal deaths and one in thirteen children in sub-Saharan Africa die before their fifth birthday [3, 4].

Failure to close the gap between current and optimal MCH outcomes within select countries is partially attributable to the poor quality of healthcare available in many resource constrained areas; in such settings, poor quality of care may prevent adequate delivery of critical services such as family planning or perinatal healthcare [5]. In healthcare service delivery, the first step towards high quality care is provider attendance [6]. Providers who are frequently absent from work present a major challenge to service delivery. Although providers may be absent from work for a variety of legitimate reasons, several prior studies have investigated or highlighted the rate, frequency, or prevalence of providers engaging in unscheduled absence from the workplace [6–13].

A prior study conducted across six countries in Asia, Africa, and Latin America found approximately 35% of healthcare providers were absent from their facility at the time an enumerator made an unannounced visit. Across all six countries, the percent of providers absent at the time of the unannounced visit ranged from 25 to 40% [6]. Across multiple prior quantitative studies, researchers have identified factors that may contribute to absenteeism within the developing country context. These contributing factors include institutional and management factors as well as community and personal characteristics [8]. At the instutitional level, contributing factors include role ambiguity, manager characteristics, human resource constraints, competing administrative duties, and the perception among providers that wages are low or insufficient [7, 8, 14]. At the community and individual level, contributing factors include negative work attitudes, low job satisfaction, competing family responsibilities, occupational burnout, and job stress [7, 8, 14, 15]. Absenteeism has been shown to be higher in smaller clinics, in areas with low infrastructure, and in situations when providers have a long commute to work [6, 10, 12]. In a report synthesizing several studies, authors found that the most common provider characteristics for absenteeism are having a higher-level cadre and authority, being male, having a greater opportunity to earn money in private practice, being posted in a poor or remote community, being posted at a lower-level health facility, and being recruited without knowing the geographical location [11]. Studies also suggest that frequent absence of higher-level staff, such as physicians, may contribute to other cadres being absent more often as well [8, 12].

Absenteeism has ramifications for MCH. It is hypothesized that absenteeism exacerbates healthcare inadequacies in developing countries and undermines demand for, quality of, and efficiency in healthcare delivery [8, 9]. When their colleagues are frequently absent, those health workers who regularly report for duty are burdened with additional work and are sometimes forced to perform for which they may be unqualified [11]. Absenteeism also has consequences for service demand, as facilities with a reputation for low reliability of services prevent mothers and children from seeking needed care [11]. More specifically, a prior study found that absenteeism contributed to women being less likely to learn their HIV status during pregnancy and less likely to deliver in a hospital or clinic [16]. Additionally, interventions that have targeted absenteeism see an improvement in both quantity and quality of health service delivery, contributing to improved child health and care-seeking for children [17, 18].

In Kenya, the country of focus for this study, a 2008 study in the Machakos district found a provider absence rate of 25% [8]. A more recent (Tumlinson et al., 2013) study using mystery clients in western Kenya uncovered multiple instances of provider absences during normal facility hours [19]. While Tumlinson et al.’s study indicates that healthcare providers in western Kenya are sometimes absent from work, it does not investigate factors that may contribute to this behavior.

While prior studies have provided an estimation of the rate of absenteeism and an understanding of associated factors, they have been primarily quantitative in nature. As such, there is limited information on the more nuanced aspects of why and how absenteeism occurs within healthcare facilities, from the perspective of those on the frontlines of service delivery. The objective of this study is to use qualitative methods to solicit nuanced healthcare provider perspectives on the scope of absenteeism within Kenyan healthcare facilities and the possible underlying factors.

## Methods

### Data collection

We conducted semi-structured interviews with 20 public and private healthcare workers in Kisumu and Nairobi. The data presented in this paper are nested within a larger study on family planning quality of care; hence these two cities were selected based on their location in provinces with modern contraceptive prevalence rates similar to the national average (53%) [20]. The study began in July 2015 with the enrollment of two initial participants, one in Kisumu and one in Nairobi. These two initial participants were identified based on their participation in prior research conducted by the principal investigator (PI; first author). The study PI asked each participant to refer one or more colleagues for possible study enrollment. Once referred, the PI then contacted each potential participant to explain the study and assess their interest and eligibility. To be eligible, participants had to be: currently employed in a health delivery role in a public or private healthcare facility that offers MCH services. All of the participants referred to the PI were eligible and consented to participate.

The study PI conducted all interviews after discussing the study protocols with eligible recruits. All of the eligible recruits consented to participate in the study via an informed consent process in which participants were told the study purpose and voluntary nature of the study and were asked for permission for tape recording. Following informed consent, service providers participated in a semi-structured interview. The semi-structured interview guide was developed by the study PI based on findings from a mystery client study conducted in Kisumu that revealed a number of negative provider behaviors [19]. Multiple researchers with expertise in family planning quality of care in low-income countries reviewed the draft interview guide and provided input; prior to implementation, two Kenyan providers reviewed the interview guide for clarity of intent and culturally appropriate language. The semi-structured interview guide consisted of questions regarding various aspects of provider motivation and provider-imposed barriers to service delivery. Information on provider motivations was obtained by asking providers to explain their reasons for becoming healthcare professionals and to provide their opinion on a number of factors hypothesized to contribute to low motivation among Kenyan healthcare providers; providers were also given the opportunity to suggest additional factors that may induce low quality of care. Information on facility-level barriers to care was obtained by asking providers if they were aware of providers – either at their own facility or in other facilities in Kenya – who engaged in behaviors such as being absent from work, soliciting informal payments from clients, or being verbally abusive to clients. To obtain a blank copy of the interview guide, see the additional material (see Additional file 1). The data presented in this article are a subset of the overall provider responses that focus specifically on the issue of provider absence.

Interviews were conducted in English (a language universally spoken by healthcare professionals in Kenya), lasted approximately 45 min, were audio-recorded, and were conducted away from the providers’ places of work where they could feel comfortable providing honest information. Providers received a small stipend to cover transportation; otherwise they were not offered any incentive for their participation. No identifying information was collected or recorded during the interview. Once all interviews were complete, they were transcribed verbatim by a professional transcriber. All transcriptions were checked for accuracy. Following transcription and data cleaning, all data were saved in Microsoft Word documents to a secure server, and the original recordings were permanently deleted from the audio recording device.

Princeton University and the Kenya Medical Research Institute reviewed and approved the study protocol and informed consent process for this study.

### Data analysis

Qualitative data analysis was performed in an iterative and collaborative process by a team of three study members. Each team member read all transcripts multiple times and wrote memos to obtain a sense of the primary study themes. Study team members convened to discuss themes and develop an initial codebook. Themes related to the interview guide questions about healthcare provider motivations and facility-level barriers to contraceptive use were sorted into thematic categories and subcategories. Study team members applied the initial codebook to a small subset of interviews. Upon completion, they reconvened to review and modify the final codebook. Examples of study codes include: absenteeism, verbal abuse, informal payments, causes of poor performance, provider motivations, and solutions. Next, each study member re-read all interviews and applied study codes to all interviews. The study team met as a group to review all coded text and to discuss and resolve any discrepancies in the coding of each of the 20 interviews. Once all interviews were coded and all study team members agreed on the coded text, study team members wrote analytic memos for each code to summarize the main findings. Nvivo software version 11 was used to apply the study codes and manage all data.

The data presented here focus on provider absenteeism. Absenteeism, in the context of this paper, refers to a situation in which a healthcare provider is not at work as scheduled for reasons *not* related to illness, scheduled vacation, or off-site work responsibilities such as professional meetings or training activities.

## Results

### Participant characteristics

Participants in the study are primarily female (*n* = 16) and work in public facilities (*n* = 13). Most respondents (*n* = 12) are nurses or community health nurses (*n* = 3). Of the remaining five participants, one is a clinical officer, two are HIV testing and counselling (HTC) coordinators, one is a health center information officer, and one is a community health worker who facilitates linkages to facility-based care. On average, participants have been providing healthcare services in the public or private sector for approximately seven years (range = one to 21 years).

### Scope of absenteeism

Half of the twenty participants report that provider absenteeism occurs frequently in health facilities in Kenya. All those who report the practice are nurses or community health nurses, with no discernable pattern related to employment sector. The most common form of absenteeism described was provider’s unannounced lateness in arriving at health facilities. Participants reported that providers may be late anywhere from 30 min to three hours which often leaves patients waiting for long periods of time unattended:

“Well, what would happen under those circumstances is that they would come to work all right, but they come late. That is if they have personal errands. They would come probably later than. They’re expected to be at work by 8:00 a.m., but this person, this provider would come in at 10:00, maybe 11:00, sometime as late as 1:00.”

- Nurse midwife, public-sector, 10 years experience.

However, absenteeism also occurs when a provider leaves the facility early, or takes an extended break in the middle of the workday. Participants noted examples of providers missing large portions of their work schedule to take an extended lunch break as noted by a public-sector community health nurse (6 years experience),“*There are times when they go for lunch, if the clients are not there, they take their time*.” In other cases, extended breaks are used to attend to personal matters, such as coursework:

“Like at times, maybe you’re at work but also in school, and you don’t want your employer to know you are in school. Because maybe your school is fulltime. So you might slip away a little bit to do one or two (school-related activities) and then come back to work.”

- Health information officer, sector unknown, 9 months experience.

Collectively, these behaviors result in reduced workdays where patients only have a limited window of time to receive care. One provider noted:

**Interviewer (I):** “How often do you think a patient goes to a facility and finds that it’s closed or that the provider is gone for the day?”

**Respondent (R):** Okay, what I can say is sometimes, we as healthcare providers sometimes - it’s not actually being closed. The facility can be open, but the healthcare provider is not in place. Maybe there’s a person in the community that went and opened the facility, but the healthcare provider reports late; the person at the facility maybe reports at, at 11 am and leaves at, at four.

**I:** Is this common?

**R:** It’s very common.

- *Nurse, private-sector, 7 years experience.*

Partial-day absence was more commonly described by participants, compared to being absent for the entire day or multiple days in a row.

Remaining participants reported that absenteeism is rare in health facilities. Providers may be absent for personal reasons related to health or errands, but this happens infrequently. A few providers mentioned that in such cases, these providers would notify their colleagues ahead of time or soon after. Among the participants who report that providers are not commonly absent, several indicated that absenteeism has been a serious problem in the past but is no longer a common occurrence. Two participants suggested the improved attendance of providers in recent years is a result of the newly decentralized health system and corresponding stricter management of public facilities. Only one participant (nurse, public-sector nurse, 5 years experience) was adamant that absenteeism never occurs. When asked about absenteeism, she responded, “*What you’re talking about is lies.*”

### Perceptions of where and why it occurs

Providers cite several factors that facilitate a culture of absenteeism among Kenyan healthcare providers, including infrequent supervision, lack of professional consequences, low accountability to patients, and insufficient wages. These different factors often intertwined creating an environment where healthcare workers can miss work with limited repercussions.

#### Infrequent supervision

Several participants attributed absenteeism to inadequate and infrequent supervision in health facilities. Providers suggested absenteeism is most prevalent in rural as compared to urban healthcare facilities because supervisory visits are rarer, “*Those facilities in the interior areas… the person in those areas can decide to report to work once in a week. There is no supervision” (*nurse, private-sector, 7 years experience)*.* Furthermore, some participants noted that there are fewer providers in rural clinics which may exacerbate the impact of absenteeism. Because of the small staff size, when one or more providers are absent, the whole clinic may be closed. As a result of being out of reach, one public-sector nurse (6 years experience) reports, the rural providers have the attitude of, “*We are the bosses here. We can do whatever we want.*”

Interestingly, one participant suggested it is easier to be absent in busy urban facilities where there are more providers (rather than small rural facilities where there may be only one provider) because in such a busy environment it is less likely for anyone to notice attendance patterns of various staff, stating: “*… but when she’s in town with a lot of staff, it’s easy for her to miss work and not be noticed* (Health information officer, sector unknown, 9 months experience).”

#### Lack of professional consequences

Providers most often reported that a lack of supervision combined with limited consequences enables absenteeism; as one participant noted,

"At the end of it all, you still get your salary… there’s no way he’s going to be reprimanded, because supervision comes once in a year… we have remote facilities in the village and the, the supervisors who are our bosses in the ministry, you know, for them to reach those facilities in the interior areas take – it’s a challenge because of maybe fuel, finances. So, the person in those – in that area can, can decide to report to work once in a week. There is no supervision.

- Nurse, private-sector, seven years experience.

In some healthcare facilities policies on absenteeism are not well enforced. Participants suggested public facilities are more vulnerable to absent staff given the differing policies for sanctioning negative behavior between public and private facility types. Some providers who worked in private facilities stated that in their experience, private facilities tend to enact immediate sanctions for those who fail to show up to work.

**R:** Yeah. Private, it’s hard. But in public, you can. But in private, it’s hard because you account for every hour that you have there.

**I**: Yeah. Sure. So you think it’s[absenteeism] very common in the public facilities.

**R:** Yeah. I guess it’s common because there is not that strictness. They are not that strict.

- Community health nurse, private-sector, 4 years experience.

Providers mentioned that it is unusual for a public-sector provider to face meaningful consequences if a supervisor discovers a pattern of absence. In rare cases, a public-sector provider may be transferred to another facility or receive a written warning.

However, it is important to note that some providers who worked in public facilities did not share the same sentiments about the lack of repercussions in public facilities. A few noted that in their facilities, absent providers are punished. One provider described how as a nursing supervisor she talks to her staff to discern why they were absent and then takes necessary actions, *“…. ‘If it’s a genuine reason, kindly, next time, try and communicate.’ Not genuine reason, just running up and down – no. You can even punish – I can even take your off. You’ll work for that day* (Nurse, public-sector, 13 years experience).”

#### Limited accountability

Additionally, participants noted that patients may be reluctant to try to report absent providers because they are unaware of confidential and reliable systems for patient feedback or complaints. Therefore, even in cases where the patient community is aware their provider is engaging in unexcused absence, “*they have nowhere to complain to”* (Nurse, private-sector, 7 years experience). Low accountability to patients combined with no supervision results in many providers feeling as if no one is watching their behavior or noting their attendance. The small patient load in some low-volume, rural facilities may lead providers to assume they won’t be missed, or that, if they are missed, they won’t be held accountable by just a few patients. A public-sector nurse (5 years experience) stated, “*Maybe they feel nobody is seeing me. I’m going to do some errand.*”

#### Low wages

Some providers felt that wages were insufficient, creating the necessity of seeking funds elsewhere to supplement low wages. As one provider notes, “*They need something for survival*.” (Community health nurse, private-sector, 4 years experience). However, numerous other providers suggested that public sector healthcare providers are paid enough for “survival,” but current wages do not reflect the skill or importance of healthcare delivery, nor do they allow for eventual upward mobility, leading providers to feel justified in looking for off-site opportunities to earn additional income during working hours. The search for additional income offsite is well illustrated by the following participant responses:

“Like you find a medical doctor, he comes to work in the morning. He’s supposed to leave in the evening. But by around midday, he’s off to another hospital to cover, because he wants that money. So he gets money from here; he’s working and it will come from another hospital. So a lot of clients, they end up not getting that quality service.”

- Information officer, sector unknown, less than one year of experience.
  “Maybe I’m working in facility A. I’m supposed to be there from 8:00 to 5:00, but I can be working from a half a day. Then in the afternoon, I can go to facility B or C to do some extra jobs so that I can earn some extra cash.”
- Nurse, private-sector, 4 years experience.

Low wages also contribute to providers feeling they are not appreciated for the considerable expertise and effort required by their job, which may reduce their motivation. In some cases, this mentality results in providers arriving late or leaving early to go look for money elsewhere. Participants described the practice of doctors or nurses seeking out “locums” at another facility. A locum is a fee provided to a healthcare professional for providing short-term services at a facility, “*Some go away to locum. When I do this I’ll get this much, instead of just sitting here waiting for the clients to come* (Nurse, public-sector, 5 years experience).” Most commonly, providers working in a public facility may also work in a private facility where wages are higher. One participant acknowledged that it is understandable to her that some doctors engage in this behavior – stating matter-of-factly, “*they have to make money”* ( Nurse midwife, private-sector, 20 years experience). But, at the same time, she’s deeply discouraged by a system that facilitates such behavior as it leaves larges sections of the population without adequate care and reserves reliable medical care for those able to pay for care at private facilities.

### The impact of absenteeism

Several participating providers expressed concern about the impact of absenteeism on Kenyan citizens in need of adequate healthcare services. One of the concerns participants mentioned was the longer wait time that provider absences, particularly late arrivals, produced:

“They are supposed to be on duty at 7:30, they handing over or they report up to 8:00, 8:30, and then they start working. But mothers will go, sit there, and these nurses will come late and start talking their stories. They are not in a hurry. And these mothers are in a hurry.”

- Nurse, private-sector, 20 years experience.

In these situations, patients incurred long wait times because they often got to the clinic much earlier than providers. Late staff, coupled with the issues of understaffing in some healthcare facilities, means that some patients either wait an extended period of time or may not even receive care on the day they went to the facility. As a result, absenteeism can have lasting effects on patient’s healthcare seeking practices by deterring people from obtaining necessary medical care in the future and doing patients an injustice:

“When a woman comes to the hospital, maybe she has left her baby in the house. So she wants to come, get that service fast, and go back. So when she comes and there’s no person to attend to her, it demoralizes her. So the next time she will think of coming to the hospital, she’ll maybe – she’ll just seek another choice of treatment.”

- Health information officer, sector unknown, 9 months experience.

“The clients are always patient. They wait for us… But we – we are not doing justice to them. But they wait until the time we come, (which) is when we start.”

- Nurse, public-sector, 10 years experience.

## Discussion

This study reports on healthcare workers’ perceptions of the scope, contributors, and impact of absenteeism in Kenyan health facilities. More than half of providers interviewed reported a belief that healthcare providers in Kenya are frequently absent from work for reasons unrelated to illness, vacation, or off-site work responsibilities. Absenteeism most commonly took the form of late arrival, early departure, or extended lunch breaks – as opposed to a full day’s absence. Unexcused absences, excessively late arrival times, and early departures from work are not acceptable practices within Kenya’s formal employment sector and the partial-day absences occur despite the clear expectation that all staff should be present from 7:30 am or 8:00 am until 5 pm. This is the first qualitative investigation into healthcare providers’ opinions on factors contributing to high rates of absenteeism within Kenya. These findings contextualize previous quantitative studies which report that absenteeism occurs frequently in health facilities and is often characterized by late arrivals or early departures [8, 19]. We found that providers attribute absenteeism to issues primarily at the institutional level and describe weak accountability systems within health facilities. Limited supervision from higher level staff paired with a lack of reliable feedback systems from patients mean that providers can be absent without repercussions. Research from Machakos, Kenya corroborates these results. Muthama et al. found that despite the high levels of provider absenteeism, there were few accounts of healthcare workers being sanctioned or fired for their excessive absences [8]. Studies in low and high income countries indicate that lack of supervision and organizational permissiveness contribute to staff absenteeism [7, 14].

Providers reported that healthcare workers are sometimes absent because they hold positions at other health facilities. The practice of dual-job holding was described as a method through which providers cope with low wages. Dual-job holding is widespread in low and middle income countries. Providers often use salaries from working in the private sector to supplement their low incomes from working at government facilities [21]. Dual-job holding can have negative impacts on public sector facilities. Providers who engage in this practice may inappropriately use public sector resources (e.g. facilities, equipment, drugs) in their private practice. They might also spend less time in the positions where they are paid least thus extending patient wait times or diverting public-sector patients into private clinics [22].

Participants disagreed as to where they believed absenteeism was most likely to occur. While most reported that public and rural facilities are more vulnerable to absenteeism, others provided counter narratives from their own experiences. Literature suggests that absenteeism is more likely to occur in public-sector clinics [9]. Some theorize that this pattern occurs because public-sector employees are guaranteed to be paid regardless of their performance [23]. In our study, one provider alluded to this saying,“… *at the end of it all, you still get your salary…*.” However, both public and private sector providers reported the behavior occurring in their facilities, and a few public-sector staff insisted that there were sanctions for absent providers in their facilities. Further, while participants insisted that absenteeism is more likely to occur in rural as compared to urban facilities, this is the opposite of what was previously found in Kenya [8]. The variation across participants may reflect heterogeneity in how individual facilities are managed. It is also possible that other influential factors may be at play including facility size, staffing, or effectiveness of clinic leadership.

Together, these findings offer a road map for potential interventions to reduce absenteeism. First, increasing frequency and type of provider supervision is a priority. More frequent and unannounced visits by higher level management (i.e. Ministry of Health) may increase attendance. Further, implementing daily monitoring systems can be impactful. A study in India found that giving teachers small incentives to track their attendance through photographs at two agreed upon times during the day led to a 50% decrease in teacher absenteeism [13]. Second, efforts should be made to open up channels for patients to provide feedback to providers. In Uganda, an intervention was conducted wherein communities were encouraged to become involved in the state of healthcare service delivery and to hold providers accountable for their performance. Results showed improvements in health service utilization and child health outcomes [17]. With respect to wages, while increasing provider salaries to reduce dual-job holding could have significant effects on providers’ attendance and motivation, such a solution may not be entirely feasible in this context. However, numerous development agencies have encouraged greater use of performance-based financing (PBF). PBF is a means of tying incentives to the performance of service providers. Those providers or facilities that meet targets or achieve high quality service delivery may receive a financial payment, bonus, or other type of incentive. In theory, PBF could create a disincentive for providers to be absent, as high rates of absenteeism among providers would reduce the total number of patients seen and lower the amount of funding available to the provider or the facility. Additional research is needed to better understand the potential impact on absenteeism of a performance pay intervention. Finally, the provision of non-monetary incentives has shown some promising effects on improving provider job performance in low-resource settings, and could be utilized to improve attendance [24].

The present study had several limitations. Providers were recruited from two large urban centers in Kenya, thus findings may not be transferable to rural locations. The study sample was also primarily composed of nurses and lower level health staff. Previous studies indicate that absenteeism is more likely to occur among higher level healthcare staff [8, 12]. Our findings may underestimate the scope of absenteeism especially as it pertains to provider cadre. Additionally, recruitment by referral can sometimes result in similar viewpoints across participants; however, the diversity of perspectives within our sample regarding the prevalence of absenteeism suggests the recruitment method did not necessarily restrict the sample to those with similar viewpoints. It is also possible that providers were reluctant to discuss negative behaviors out of fear of presenting themselves or their colleagues in a negative light or due to concerns that such reports could be misconstrued as personal admission of being absent. This could result in further underestimation of the scope of absenteeism. Finally, the qualitative study design – which utilized purposeful sampling techniques in order to select information-rich cases – limits statistical generalizability. However the primary findings that absenteeism in Kenya impacts healthcare access and is underpinned by institutional-level issues such as infrequent supervision and low wages resonates with a larger body of literature on other negative provider behaviors such as informal payments and disrespectful treatment.

## Conclusion

Provider absenteeism puts considerable strain on patients, clinic resources, and other staff in already resource-limited settings. We found that provider absence occurs in a variety of health facilities in Kenya, and is linked to institutional-level issues. More research is needed to assess the prevalence of absenteeism on a national or subnational level and more formative research is needed to develop and understand the feasibility and sustainability of promising interventions.

## Additional file

Additional file 1: Semi-structured Interview Questions. (DOCX 15 kb)

## Data Availability

The datasets used and/or analysed during the current study are available from the corresponding author on reasonable request.

## Abbreviations

HTC: HIV Testing and Counseling
MCH: Maternal and Child Health
NICHD: National Institute of Child Health and Human Development
NIH: National Institutes of Health
PI: Principal Investigator
