# Supplementary material for: Understanding healthcare provider absenteeism in Kenya: a qualitative analysis
Source: BMC Health Serv Res. 2019 Sep 11;19:660. doi: 10.1186/s12913-019-4435-0 (PMC6740012; doi:10.1186/s12913-019-4435-0)
Supplement: Supplementary file 1 — Semi-structured Interview Questions. (DOCX 15 kb) [file 12913_2019_4435_MOESM1_ESM.docx]

**Qualitative Study on Family Planning Service Quality**

Semi-structured Interview Questions

1. What is your cadre and how long have you worked as a health care provider in Kenya?
2. Can you tell me what you do on a typical day at work in your present department?
3. Can you please tell me about the last time you observed or heard about a woman who came to a health care facility seeking reproductive health care services such as family planning counseling or care during labor and delivery but was denied services by a health care provider?
4. Is it common for health care providers to be absent from work?
   1. How often do providers miss work for reasons unrelated to illness or off-site responsibilities?
   2. How often, in your opinion, does a client arrive at a health facility during working hours to find the facility is closed?
5. Do some service providers ask clients to pay for services or commodities that are supposed to be provided for free?
   1. If so, how common is this practice?
   2. Why do you think providers take money from clients in this way?
   3. Do you think this practice should be stopped and, if so, how can it be stopped?
6. What are the biggest barriers Kenyan women face when they arrive at a health care facility and are in need of reproductive health care services?
7. What are some ways to address or overcome these barriers? Is better supervision a possible solution?
8. Why do you think some providers are less motivated than others to do their job well? (Probe: In other words, why do some providers say they have a “calling” to provide health care or appear to be concerned about the well-being of their patients while other providers do not appear concerned about the interests and well-being of their patients?)
9. What can be done to help more providers feel motivated to do their job well?
10. Do low wages result in low provider motivation? (Probe: tell me more)
11. Could high numbers of patients and few staff result in low provider motivation? (Probe: tell me more)
12. Could lack of electricity, running water, and critical medical supplies result in low provider motivation? (Probe: tell me more)
13. How do corrupt practices, such as bribes, contribute to poor delivery of health care services? (Probe: in what way?)
14. Is there any punishment for providers who turn away clients, offer poor quality services, are frequently absent, or who take money from clients? For example, is a provider likely to be fired for one of these reasons or reassigned to a less desirable location?
15. What types of supervisory practices and policies are needed to improve service delivery?
16. Is there anything you would like to add?
17. Do you have any questions for me about me or this study?
